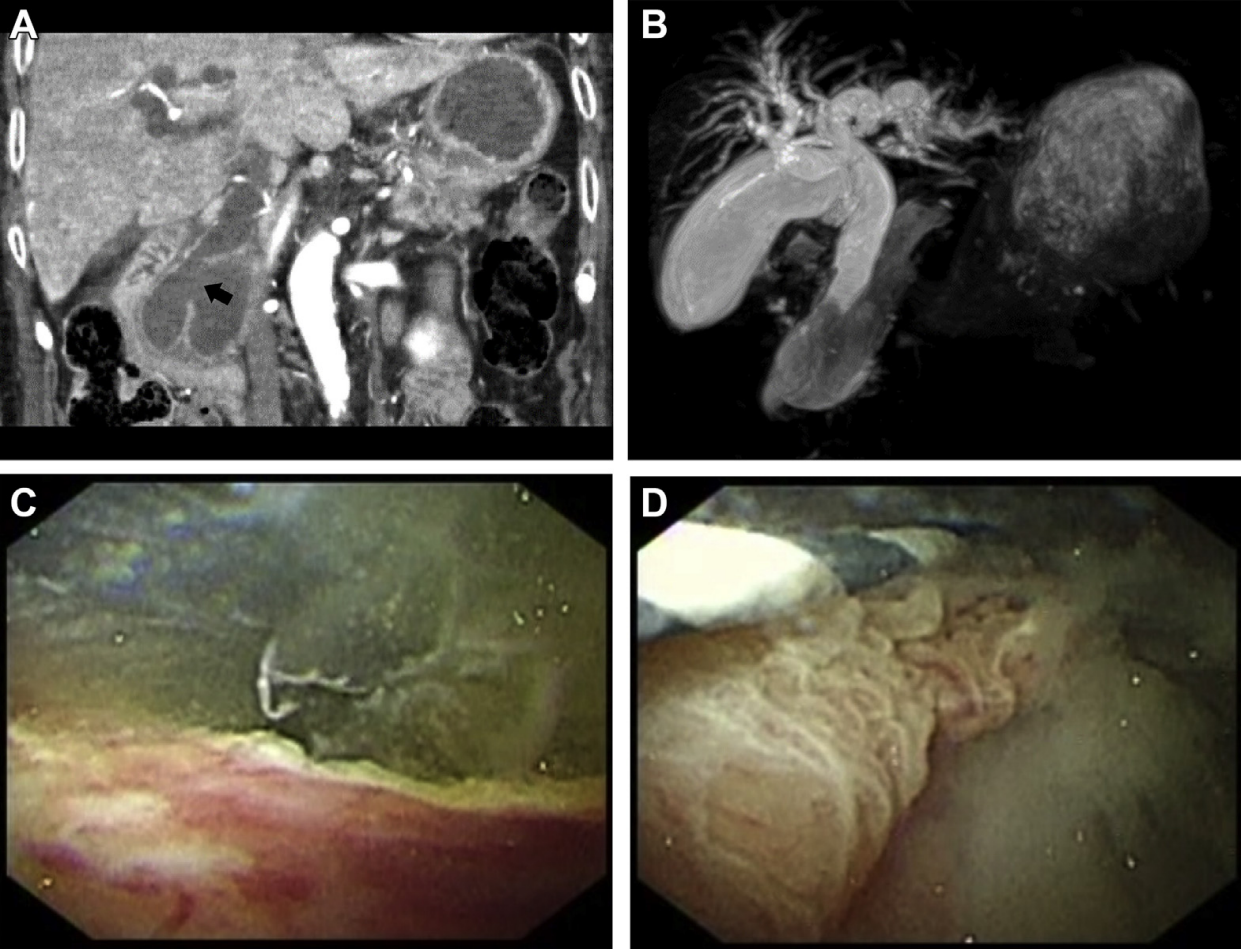# Digital Cholangioscopy Visualized a Pancreaticobiliary Fistula Associated With Intraductal Papillary Mucinous Neoplasm

**DOI:** 10.1016/j.gastha.2021.09.009

**Published:** 2022-02-03

**Authors:** Tsuyoshi Suda, Ayaka Nanbu, Naoki Oishi

**Affiliations:** Department of Gastroenterology, Kanazawa Municipal Hospital, Kanazawa, Ishikawa, Japan

An 83-year-old woman presented with jaundice at our hospital. Contrast-enhanced computed tomography revealed a large intraductal papillary mucinous neoplasia (IPMN) with ectasia of the main pancreatic duct and intrahepatic bile ducts. An inferior common bile duct pancreaticobiliary fistula was also detected (Figure A: arrow). Subsequently, magnetic resonance cholangiopancreatography showed signal changes in the region, signifying massive amounts of mucus (Figure B).

Egress of mucus was visualized on endoscopy from a dilated ampullary orifice. We attempted to observe the pancreaticobiliary fistula via digital cholangioscopy (CHF-V2; Olympus, Tokyo, Japan). Copious mucous flow from the pancreaticobiliary fistula was observed at the lower portion of the common bile duct (Figure C). This fistula’s location was ascertained, and its edge commenced at an ulcerating lesion (Figure D).

Despite efforts, the patient died from obstructive jaundice and recurrent cholangitis. The main pancreatic tumor was confirmed to be an IPMN on autopsy, and there was no evidence of malignancy. It was surmised that a pancreaticobiliary fistula arose from perforation because of increased intraluminal pressure from mucus production of the IPMN.

Digital cholangioscopy remarkably enhanced visualization in this case and is becoming widely used for diagnosis and treatment of biliary lesions. Such direct observation is exceedingly rare.

**Figure:**